# Post-Infarction Ventricular Septal Rupture Complicated by Cardiogenic Shock Requiring Mechanical Circulatory Support as a Bridge to Definitive Therapy During the COVID-19 Pandemic

**DOI:** 10.7759/cureus.16421

**Published:** 2021-07-16

**Authors:** Ciril Khorolsky, Dyer Pettijohn, Neil Yager

**Affiliations:** 1 Cardiology, Albany Medical Center, Albany, USA; 2 Internal Medicine, Albany Medical Center, Albany, USA; 3 Cardiology, Albany Medical College, Albany, USA

**Keywords:** ventricular septal rupture, mechanical circulatory support, stemi, va-ecmo, covid-19

## Abstract

Ventricular septal rupture (VSR) is a devastating complication of acute myocardial infarction (MI) and is often associated with cardiogenic shock. Although considered to be very rare in the reperfusion era, recent reports have demonstrated an increased frequency of post-MI VSR cases during the COVID-19 pandemic. Despite advances in surgical repair and management strategies over the past decades, mortality rate has remained high, especially in hemodynamically unstable patients. In VSR patients with cardiogenic shock, the use of mechanical circulatory support (MCS) could be used as a bridge to surgical intervention. We report a patient with VSR complicated by cardiogenic shock secondary to late presenting MI, managed successfully with venoarterial extracorporeal membrane oxygenation (VA-ECMO) as a bridge to surgical intervention.

## Introduction

Ventricular septal rupture (VSR) is a rare but life-threatening complication of acute myocardial infarction (MI) often leading to hemodynamic instability and cardiogenic shock. It is associated with very high morbidity and mortality, especially in the setting of cardiogenic shock [1] with a reported survival of less than 10% at one month without surgical repair [2]. Compared to the pre-reperfusion era, the incidence of post-MI VSR has progressively declined with the advent reperfusion strategies to less than 0.5%, with more recent analysis estimating an incidence in the range of 0.17%-0.21% [3,4]. During the COVID-19 pandemic, an increased incidence of MI-related mechanical complication was observed despite a reduction in the number of acute MI presentations [5-7]. Surgical intervention remains the treatment of choice, however, the rarity of VSR in the era of reperfusion therapy along with the controversies regarding the optimal timing for surgery have resulted in lack of medical and surgical expertise in the management of VSR [8]. We present a patient with post-MI VSR complicated by cardiogenic shock successfully treated with the use of venoarterial extracorporeal membrane oxygenation (VA-ECMO) as a bridge to surgical intervention.

## Case presentation

**Figure 1:**
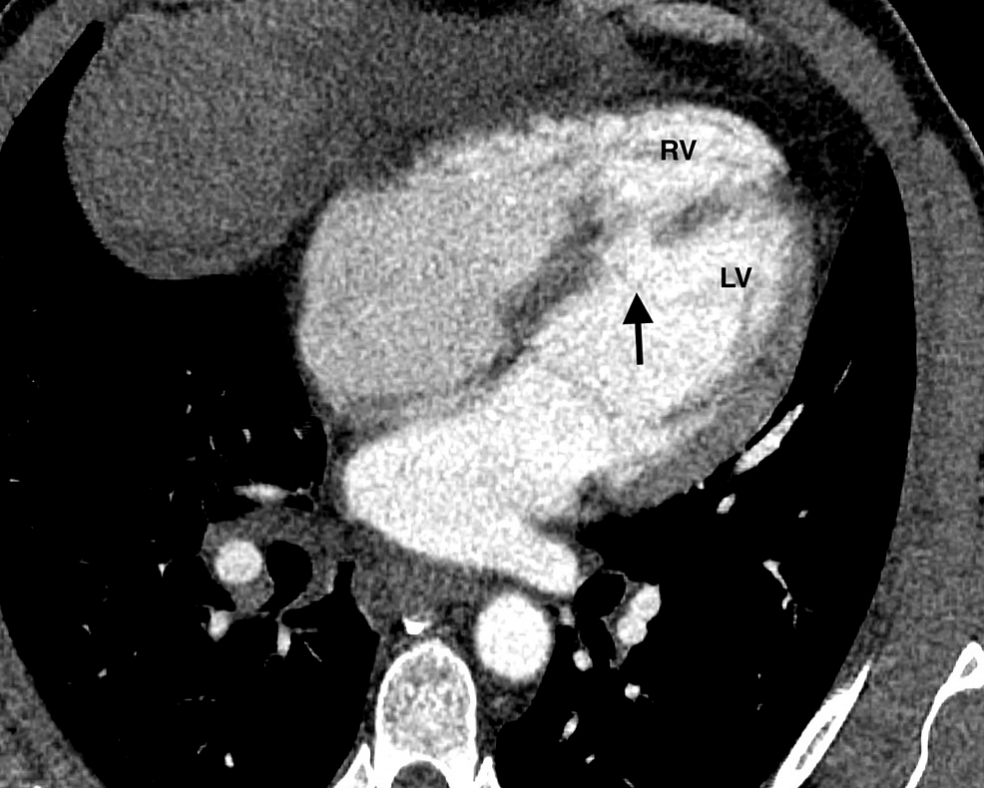
Chest computed tomography angiography demonstrating a large ventricular septal rupture in the mid inferior septal wall (arrows) with free communication between the right and left ventricles. LV, left ventricle; RV, right ventricle.

A 50-year-old male presented to an outside hospital with one-day of worsening abdominal pain and a reported episode of chest pain associated with profuse diaphoresis eight days prior to presentation. Computed tomography angiography (CTA) of the chest revealed a large inferior mid-septal myocardial wall defect (Figure 1).

**Figure 2:**
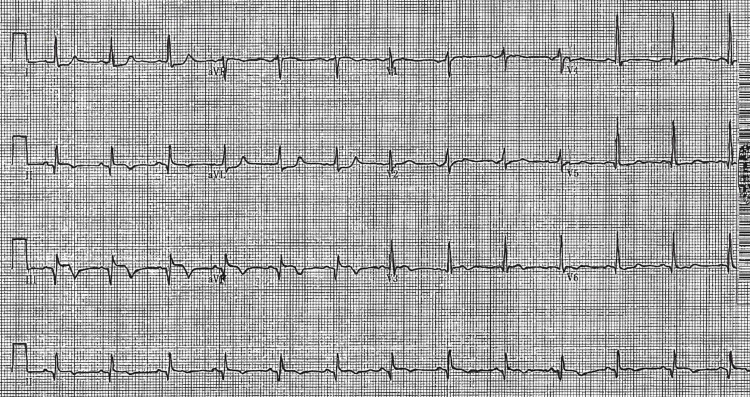
12-lead electrocardiogram demonstrating ST elevations in leads II, III and aVF with associated Q waves and reciprocal ST depressions in leads I and aVL.

Subsequently, the patient was transferred to our hospital for urgent intervention. Upon arrival, the blood pressure was 79/66 mmHg with a heart rate of 104 beats per minute. Auscultation noted a 3/6 holosystolic murmur at the left sternal border and an electrocardiogram (ECG) demonstrated normal sinus rhythm with inferior ST-segment elevations with Q waves (Figure 2).

**Figure 3:**
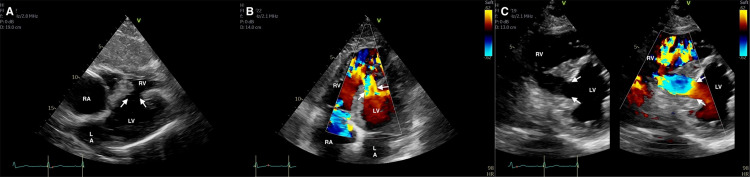
Transthoracic echocardiography. (A) Subcostal view of the ventricular septal rupture in 2D (arrows). (B) Apical four-chamber view of the ventricular septal rupture with left-to-right shunting on color doppler (arrows). (C) 2D with color flow comparison in the parasternal short-axis view demonstrating a large VSD with a significant left-to-right shunt (arrows). LA, left atrium; LV, left ventricle; RA, right atrium; RV, right ventricle.

Laboratory data was notable for elevated troponin I of 2.3 mg/dL (0.00-0.04 mg/dL), creatinine of 2.12 mg/dL (0.80-1.4 mg/dL), aspartate aminotransferase (AST) of 974 IU/L (5-45 IU/L), alanine aminotransferase (ALT) of 707 IU/L (5-60 IU/L) and lactic acid of 9.84 mmol/L (0.4-2.0 mmol/L). He was loaded with Aspirin and started on Norepinephrine with Dobutamine due to hemodynamic instability with end-organ damage. Transthoracic echocardiography showed a large VSR in the mid-inferior septal wall with a significant left-to-right shunt. Left ventricular (LV) diastolic dimension was normal with an estimated LV ejection fraction of 65%. The right ventricle (RV) was noted to be dilated with hypokinetic RV free wall and severe tricuspid regurgitation (Figure 3).

**Figure 4:**
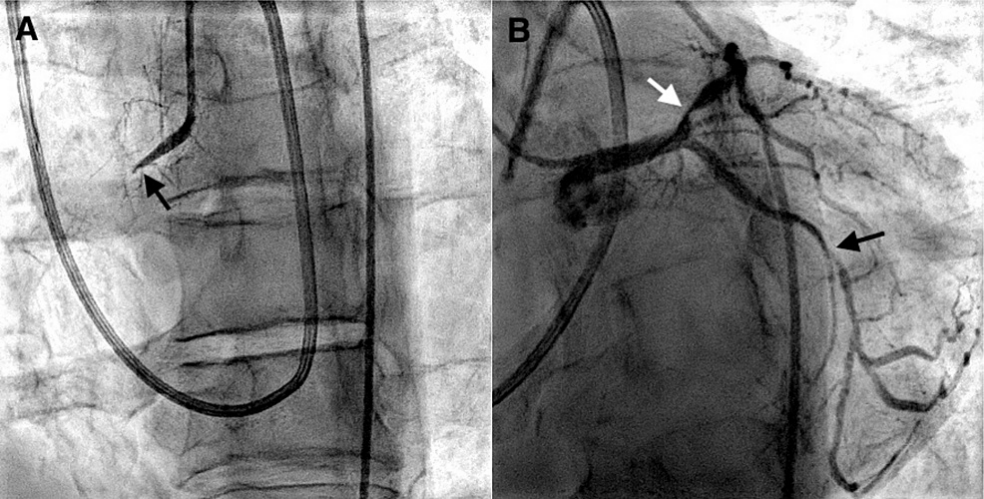
Coronary angiography. (A) Culprit 100% occlusion of the proximal RCA (black arrow). (B) 90% stenosis in the proximal LAD (white arrow) and 80% stenosis in the second OM (black arrow). RCA, right coronary artery; LAD, left anterior descending artery; OM, obtuse marginal.

The patient underwent urgent coronary angiography which revealed culprit 100% occlusion of the proximal right coronary artery with 90% stenosis in the proximal left anterior descending artery and 80% stenosis in the second obtuse marginal off the left circumflex artery (Figure 4).

An emergent surgical repair of VSR was considered, however, due to worsening cardiogenic shock with multisystem organ failure despite escalating doses of vasopressors and inotropic support, the patient was deemed too high of a surgical risk. Therefore, it was decided to implant a peripheral VA-ECMO system to stabilize the patient and serve as a bridge to surgical intervention. On hospital day three, he underwent VSR repair and coronary artery bypass grafting (CABG) with a left internal mammary artery to left anterior descending artery, and saphenous vein graft to the second obtuse marginal. Postoperatively, he developed severe biventricular failure and required implantation of a biventricular assist device, which was further complicated by mediastinal bleeding. The patient was subsequently listed for a heart transplant and eight weeks later he underwent successful orthotopic heart transplantation without any complications. The patient has had an uneventful post-transplant course and continues to do well at present.

## Discussion

VSR remains a lethal mechanical complication of acute MI, although rare it is well recognized and is often associated with cardiogenic shock. It usually occurs within the first 24 hours or three to five days post-infarction, rarely occurring even later, and has a variable clinical presentation ranging from complete hemodynamic stability to total cardiovascular collapse [2,4]. Risk factors associated with the development of post-MI VSR include older age, female gender, prior stroke, chronic kidney disease, ST-segment elevation, elevated cardiac markers, higher Killip class, tachycardia, hypotension and delayed or lack of reperfusion [4,9]. Post-MI VSR results in left-to-right shunting, right ventricular (RV) volume and pressure overload, increased pulmonary venous return, and secondary left-sided volume overload, ultimately leading to biventricular failure. The clinical effect varies depending on the size of the defect, its expansion over time, infarct area, degree of shunting, and severity of RV dysfunction. Nonetheless, the majority of patients develop hemodynamic deterioration in the hours or days following VSR [4,10].

The management of post-MI VSR is challenging and is associated with significant postoperative mortality [11]. Surgical repair is considered the treatment of choice, however, the optimal timing of surgery remains controversial, especially among hemodynamically unstable patients [10]. While the 2013 American College of Cardiology and American Heart Association Guidelines recommend emergent surgical repair in all VSR patients irrespective of hemodynamic status [12], a growing body of published data suggests improved survival with delay in VSR repair. In a 2005 study of 189 patients undergoing surgical repair of post-MI VSR, Jeppsson et al. demonstrated a three-fold increase in survival in patients who underwent surgical repair after 72 hours following presentation, compared to surgical repair within 24 hours [13]. Similarly, in a 2012 analysis of surgical outcome in 2876 VSR patients, Arnaoutakis et al. found that patients who underwent repair within the first 24 hours had the highest mortality, with a significant decrease in mortality for patients whose surgical repair was delayed. The reported 30-day mortality was 54.1% with surgical repair in the first seven days from MI compared to 18.4% for patient undergoing repair after seven days [14].

The improved outcome with delayed surgical correction may be related to post-infarction physiologic tissue remodeling. In the acute setting, infarcted myocardium is weak, friable and hold sutures poorly. This may lead to impaired healing with increased risk of tearing and residual shunt formation [10]. Delaying repair allows for new collagen deposition, which usually begins by days 2-4 and completely replaces all necrotic myocytes by 28 days post-MI, thereby allowing friable tissue to strengthen, which may facilitate the VSR repair [15]. Nonetheless, in hemodynamically stable patients, early surgical repair should be considered due to the risk of abrupt VSR expansion which may result in sudden hemodynamic collapse in previously stable patients [1,16]. In hemodynamically unstable patients, an early surgery approach may be detrimental and is associated with very high mortality [1,2]. This is reflected in the 2017 European Society of Cardiology guidelines, which promote delayed elective repair in patients initially responding to aggressive conservative therapy [17].

Mechanical circulatory support (MCS) has become a useful tool in the management of VSR patients in cardiogenic shock. By augmenting cardiac output, improving systemic perfusion, and reducing shunting MCS allows for hemodynamic stabilization prior to definitive treatment [10,16]. Intra-aortic balloon counterpulsation (IABP) has been used extensively in unstable VSR patients and functions by reducing afterload, decreasing left-to-right shunting, increasing coronary flow, and reducing ventricular wall stress and oxygen demand [10]. However, it does not provide sufficient hemodynamic support and is associated with clinical deterioration when used as a single device [16]. In recent years, VA-ECMO has emerged as an effective option, often superior to IABP, which provides adequate hemodynamic support as well as a bridge to definite surgery [10,16]. The use of VA-ECMO in VSR patients is not without its limitations, which include bleeding and increased afterload resulting in higher myocardial oxygen demand that may impede ventricular recovery. Additionally, the increased ECMO blood flow and afterload may worsen left-to-right shunting leading to RV dysfunction and higher risk of VSR expansion [16,18]. As it currently stands, per the recent European Society of Cardiology Guidelines, it is a class IIa recommendation to use short-term MCS as bridge to recovery or surgery in VSR patients with persistent cardiogenic shock [19].

## Conclusions

VSR remains a fatal complication of acute MI often associated with refractory cardiogenic shock. Although rare in the era of reperfusion, while the COVID-19 pandemic continues, physicians should be prepared to encounter this complex complication. In post-MI VSR patients with hemodynamic instability, the use of VA-ECMO appears to be a viable management strategy for hemodynamic stabilization and as a bridge to definitive therapy.
